# Leadership, governance and management for improving district capacity and performance: the case of USAID transform: primary health care

**DOI:** 10.1186/s12875-020-01337-0

**Published:** 2020-12-04

**Authors:** Binyam Fekadu Desta, Azeb Abitew, Ismael Ali Beshir, Mesele Damte Argaw, Sualiha Abdlkader

**Affiliations:** USAID Transform: Primary Health Care, JSI Training & Research Institute Inc. in Ethiopia, P.O. Box 1392, 1110 Addis Ababa, Ethiopia

**Keywords:** Leadership, Management and governance, District health management, Health system performance, Primary health care

## Abstract

**Background:**

Primary health care (PHC) in Ethiopia serves as the main entry point for preventive, promotive and curative health services. The district health office is responsible for the planning, implementation and evaluation of all district health activities. In addition, district health offices manage service delivery facilities working on provision of PHC – primary hospitals, health centers and health posts. As the leader of the health care system tier, district health management must ensure direction, alignment and commitment within teams and organizations and make sure that achievements are consistent with the vision, values and strategy of the organization. USAID Transform: Primary Health Care provides diverse support to improve district health manager competencies including in-service trainings followed by planning and implementation of performance improvement projects and on-the-job mentoring and support.

**Methods:**

This study was conducted to compare district level capacity and performances between leadership, management and governance (LMG) and non-LMG districts. Project outcome monitoring data that shows the performance of districts was collected from 284 districts from January to December 2019. The study was carried out using a comparative-cross sectional study design, which assessed and compared district health office level indicators. Districts were classified into two categories: LMG and non-LMG districts. The study compared data from 94 LMG and 190 non-LMG districts. Propensity score matching was used to control the effect of differences between LMG and non-LMG districts.

**Results:**

Results of the independent samples t-test revealed that LMG districts scored better average performances of 61.8 ± 121.45 standard deviation (SD) compared to non-LMG districts 56.89 ± 110.39 SD, with t (282243) = − 3.407317 and *p* < 0.001, two-tailed. The difference of 4.9 percentage unit in the average performance indicated a statistically significant difference between the LMG and non-LMG districts.

**Conclusion:**

District level leadership development program contributes to improving district capacity, structure and management practices, and quality of care.

**Supplementary Information:**

The online version contains supplementary material available at 10.1186/s12875-020-01337-0.

## Background

The World Health Organization (WHO) and its member states have endorsed primary health care (PHC) in the Alma Ata declaration. In the declaration, PHC is stated as, ‘an essential health care based on practical, scientifically sound and socially acceptable methods and technology, universally accessible to individuals and families in the community through their full participation and at a cost that the community and country can afford to maintain at every stage of their development in the spirit of self-reliance and self-determination’ [1]**.** The government of Ethiopia has long been implementing the principle of primary health care in the country. Primary health care in Ethiopia serves as the main entry point for preventive, promotive and curative health services. The district health office is responsible for the planning, implementation and evaluation of all district health activities. In addition, district health offices manage service delivery facilities under PHC – primary hospitals, health centers and health posts. In its strategic plan for the period between 2015 and 2020, the government of Ethiopia has outlined achieving excellence in leadership as a priority area [2]. Availability and effective use of resources, appropriate planning and management of primary care is important for quality, cost-effective and equitable health service delivery. Moreover, trained and motivated health workforce including managers are vital to appropriate functionality of PHC [3].

As overseers, district health management are responsible for overall functioning of primary health care including planning, performance review and management, effective use of resource and monitoring and support for health service delivery. Furthermore, district management staff need to have skills in strategic problem solving, human resource management, financial management and operations management which enables and facilitates strong management at the facility level as well as create well-functioning facilities and improved health outcomes [4]. A leader at the district health service has to ensure direction, alignment and commitment within teams and organizations and make sure that achievements are consistent with the vision, values and strategy of the organization [5]. Effective leaders promote continuous development of knowledge, skills and abilities of staff in order to improve quality of service delivery and consistently encourage and motivate changes and deal with poor performance [6]. According to the Federal Ministry of health (FMOH), management and governance within the health sector shows critical gaps that include ‘the capacity to implement health care; improve the utilization of health services; systematically follow-up on the implementation of policies, guidelines, standards and protocols; implement reforms in a timely manner and enhance the coordination of public-private partnerships’ [2].

There is evidence suggesting that empowering leaders and mangers with required principles and competencies leads to improved health system performance through enhanced work environment and strong management systems which will eventually create responsive health system with improved allocation of resources [7]**.** Consequently, the FMOH has introduced an in-service training program on Leadership, Management and Governance (LMG) in 2017 [8].

The introduction of LMG in-service trainings in Ethiopia resulted in significant improvement in service delivery and utilization, work environment, provider motivation, teamwork and resource management. This study also revealed that implementation of LMG training followed by supervision and monitoring resulted in improved implementation of LMG related functions, improved capabilities and initiated a sense of positive competition among health facilities to achieve planned facility-level service delivery objectives [9]**.** Like this finding - in other countries - in-service training has been shown to empower leaders and managers to appropriately implement district health activities in an organized way through sound management of primary health services, team building, and supervision. In addition, in-service training for managers is a significant investment in creating and maintaining critical competencies required for district level service settings [10].

USAID Transform: Primary Health Care supports the government’s strategic initiatives in 117 primary hospitals, 1856 health centers, and 9291 health posts in over 400 districts through the provision of phased and adaptive technical assistance. It also integrates leadership, management and governance practices as a key part of the Activity’s components to create a capacitated management at the primary health care level to face challenges and garner improvements in service delivery and performance. Leadership, management and governance (LMG) capacity building is mainly about addressing the gaps that exist in managers and health workforces at the primary health care level so that they can facilitate equity and quality health services. This study thus aimed to compare capacities and performances between LMG and non-LMG districts.

## Methods

### Study setting

Ethiopia has  ten administrative regions and two city administrations. Each of these ten regions is divided into zones and each zone is divided into districts. A district health office is available at the lower level of the administrative structure and is a key driver of primary health care performance. On average, one district is expected to support 20 health posts, four health centers and a primary hospital.

### Intervention

The Activity uses various approaches including provision of leadership, management and governance trainings at the district level with major contents of overview and context of the Health System in Ethiopia; Introduction to Leadership, Management and Governance; Improving Performance through enhanced Leadership, Management and Governance; Resources Management; and Health Service Delivery Management. The training approach is team-based and experiential learning which entails including two to three people from each district and allowing open discussion to share experiences among themselves. Until the end of the session, these participants are considered as a working team. It also trains managers from district health offices, primary hospitals and health centers and then they will support the leadership at the primary health care level to strive for change by implementing leadership projects. The projects are linked to maternal and child health related issues. One of the woredas, for example, aimed at improving EPI data quality from the current 23 to 95% by the end of April 30,2020. Moreover, following capacity building training, trainees must organize on the job orientation for their colleagues in order to create same level of understanding and thus work together towards the same goal. The trained people with their counterparts in their facility work together to scan their current situation, design performance improvement projects, identify their stakeholders and mobilize resources and jointly conduct monitoring & evaluation. Onsite coaching and technical support are also provided by LMG trainers, project staff, and Zonal Health Department staff using a standard coaching checklist and following OALFA (Observe, Ask, Listen, Feedback, and Agreed) technique. In addition, learning sessions are organized through performance review meetings (PRM) to share challenges, and success and lessons at different levels.

### Data source

USAID Transform: Primary Health Care works towards improving public health service delivery and management. As part of this effort, one of the major interventions is supportive supervisions to its intervention district health offices with the objective of providing onsite technical support and producing program outcome monitoring data. A supportive supervision checklist is a set of questions related to reproductive, maternal and child health and health system interventions which was developed by the USAID Transform: Primary Health Care Activity to guide field level support. The checklist is organized to frame a two-way discussion between the supervisor and the health worker at each district health office. Each question has a definition, decision point and a response documentation section to inform improvement plans. This study employed the supportive supervision program outcome monitoring data collected from 284 district health offices during the January to December 2019 fiscal year.

### Study design and instruments

The study was carried out using a comparative-cross sectional study design, which assessed and compared district health office level indicators. Districts were classified into two categories: LMG and non-LMG. The study compared data from 94 LMG and 190 non-LMG districts. In this study, LMG districts were districts with at least one LMG trained staff that were implementing the LMG health service management improvement projects. The supportive supervision checklist is comprised of 94 questions and more than 75% of the questions are binary i.e. seeking ‘true’ or ‘false’ responses. For assessment purposes, the checklist was categorized into five major domains focusing on the interventions of the Activity. A total of 44 questions were arranged into five categories: district capacity, service availability, resource mobilization and use, quality of services, and management practices and structure (Additional file 1). All the 44 questions categorized were binary (true or false) response types.

### Data collection

During district health office support, data collection and entry were conducted onsite using an online electronic system and tablets. The system allows the questionnaires to be programmed and follows skip patterns based on previous responses. The district health office support, data collection and entry were done by the project cluster offices staff. On a few occasions, the visit may be carried out by other experts from regional project offices who use paper and then transfer the data to the online system. Data were collected by interviewing the district managers and reviewing all relevant documents.

### Data analysis

Data were managed using a web-based system, District Health Information System (DHIS2) [11], and exported to Statistical Package for Social Sciences (SPSS) version 25 for statistical analysis. Propensity Score Matching (PSM) analysis was conducted using R-plugin for propensity score matching for SPSS.

Propensity score matching was used to explain possible differences in the baseline variables between LMG and non-LMG districts. Propensity score matching is a tool to adjust a treatment effect for measured confounders in non-randomized studies [12]. The logic behind propensity score methods is that balance on observed covariates is achieved through careful matching on a single score – the estimated propensity of selecting the treatment, or simply the propensity score. The propensity score is defined as the probability of receiving treatment based on measured covariates [13]. In this study, the covariates used for the calculation of the propensity score were district catchment population, number of HCs, number of HPs, number of ambulances the district has, and number supportive supervision visit frequency. A binary treatment indicator (0 for non-LMG\control and 1 for LMG\treatment), nearest neighbor matching, caliper of 0.2, logistics regression estimation, and matching ratio of 1 to 2 were used to perform the PSM.

We checked the balance of covariates using the relative multivariate imbalance L1 test and standardized mean differences (SMD). The L1 metric is theoretically between 0 and 1. The smaller the L1 metric, the better the matching result [14]. Differences in the distribution of observed district characteristics between the LMG and non-LMG groups in the unmatched and matched samples were compared using SMD. We considered SMD greater than 0.1 as indicative of statistically significant difference, and vice-versa. We did not use Chi-square or the Student’s t-tests to compare differences in baseline characteristic because the associated *p*-values are sample size dependent. Accordingly, these tests are not recommended for checking covariate balance [15].

Finally, the matched data after PSM were used to analyze and evaluate the intervention effect with the reduction of confounding bias due to PSM. Accordingly, the mean and standard deviations were calculated for all five categories and the differences in mean scores of district capacity, service availability, resource mobilization and use, quality of services, and management practices and structure between LMG and non-LMG districts were examined using independent samples t-test and Levene’s test, with the level of significance being determined at a *p*-value < 0.05.

### Ethical clearance

This report used project data that has been collected as part of the follow-up monitoring visit to district health offices. Before the data collection, verbal consent was taken from each participant. The results of the study did not distinguish the name of the district and other specific site identifiers. Therefore, JSI research and Training Institute, Inc’s Institutional Review Board (IRB) has determined that this activity is Exempt from human subjects’ oversight (IRB #20-15E).

## Results

### PSM and characteristics of the study districts

A total of 284 districts, 94 LMG and 190 non-LMG, were included in the study. The propensity matching process yielded groups that were well matched based on the characteristics of the districts (Table 1). In the propensity score model, we were able to match 88 LMG districts to similar controls group of 157 non-LMG districts. The relative multivariate imbalance L1 was 0.910 before matching and 0.909 after matching. There were no covariates exhibited large imbalance of |d| > 0.25.

**Table 1:** Districts characteristics before and after PSM

	Before Propensity Score Matching			After Propensity Score Matching		
	LMG Districts Means	Non-LMG Districts Mean	Std. Mean Diff.	LMG Districts Means	Non-LMG Districts Mean	Std. Mean Diff.
N	94	190		88	157	
Propensity Score	0.344	0.325	0.262	0.337	0.333	0.046
Catchment population	134,461.8	127,487.2	0.123	132,360.6	129,554.0	0.050
# of HCs	5.1	4.9	0.092	5.0	5.0	−0.003
# of HPs	26.0	24.9	0.110	25.7	25.7	−0.005
# of ambulances the district has	2.7	2.5	0.129	2.6	2.6	0.011
# of supportive supervision visit frequency	2.1	1.9	0.213	2.0	1.9	0.034

Before PSM, nearly all the characteristics of the districts varied considerably between the LMG and non-LMG groups with SMD exceeding the 0.1 limit. The LMG and non-LMG groups were only similar with respect to number of HCs (SMD = 0.092) since the SMD was less than 0.1. However, after matching districts in the non-LMG group to those in the LMG group on similar propensity scores, all the characteristics became similar between districts in the LMG and non-LMG groups (all SMD was less than 0.1).

### Comparison of LMG and non-LMG districts after PSM

The comparison statistics considered the matched data after PSM. Levene’s test for equality of variances assessment revealed that there was homogeneity of variance among the five categories and average performance (Table 2). Therefore, an independent t-test was run on the data with a 95% confidence interval (CI) for the mean difference. Results of the independent samples t-test revealed that LMG districts showed better average performances 61.8 ± 11.5 standard deviation (SD) compared to non-LMG districts 56.9 ± 10.9 SD, with t (243) = − 3.317 and *p* < 0.001, two-tailed. The difference of 4.9 percentage point in the average performance indicated a statistically significant difference between the LMG and non-LMG districts.

**Table 2:** Levene’s Test for Equality of Variances

	F	Sig.
Management practices and structure	0.248	0.619
Quality of services	0.140	0.709
Resource mobilization and use	0.342	0.559
Service availability	0.250	0.618
District capacity	1.422	0.234
Average Performance	0.134	0.714

There were statistically significant differences between LMG and non-LMG districts in three items on the five categories with *p*-value < 0.05 (Table 3). These were noted in categories that addressed management practices and structure, quality of services, and district capacity.

**Table 3:** LMG and Non-LMG districts comparison

		Mean	SD	t	*p*-value
Management practices and structure	Non-LMG	56.7	19.2	−2.789	0.006^*^
	LMG	63.8	19.0		
Quality of services	Non-LMG	54.2	20.2	−2.155	0.032^*^
	LMG	59.9	18.9		
Resource mobilization and use	Non-LMG	56.3	17.4	−1.910	0.057
	LMG	60.9	18.7		
Service availability	Non-LMG	65.0	9.6	−0.538	0.591
	LMG	65.7	9.7		
District capacity	Non-LMG	52.2	18.3	−2.553	0.011^*^
	LMG	58.7	20.9		
Average Performance	Non-LMG	56.9	10.9	−3.317	0.001^*^
	LMG	61.8	11.5		

There were no significant differences in the resource mobilization and use and service availability categories between the two groups data.

## Discussion

The finding of the study suggests that implementing LMG activities significantly improves management practices and structure, quality of services, and district capacity. The study indicates that district health managers’ performance is highly related to performance of other primary health care entities. This finding is consistent with other similar research in other settings where improving performance of district management improved the performance of primary health care functions [16, 17]. The analysis of district management administrative capacity shows differences between LMG and non-LMG districts. The study reveals differences between LMG and non-LMG districts in capacitating health workers, strengthening teams and availing inputs. Using selected administrative performance indicators, the study found overall differences in the capacity created between non-LMG districts (52.2%) and LMG districts (58.7%). The improvements may be related to the practice of effective management which has direct implications in influencing availability of input and redesigning of processes [18]. This result is consistent with a study done in Ethiopia that shows improving competencies at the district level can improve service availability and quality by improving capacity of management at the facility level, coordination, resource availability and accountability [4].

The study also shows clear difference in the capacity of districts to establish multi-sectoral coordination, availability of trained health workforce to improve service quality as well as functional equipment between LMG and non-LMG districts. It found a clear difference between the two districts (non-LMG districts - 56.7% and LMG districts - 63.8%). The results of this study are similar with previous findings where capacitating management improved management practices which is attributable to improved commitment [19]. Capacity building is also thought to improve team motivation and performance of planning skills and enhances management practices [20]. Other studies also show that capacitating managers improves workplace environments, including handling human resource management, and the day-to-day activities and teamwork [21].

From a resource mobilization and use perspective, the study showed no difference between the two types of districts (non-LMG districts - 56.2% and LMG - 60.9%) in allocating budgets for key activities, implementing an effective Community Based Health Insurance (CBHI) scheme and availing and using vital resource like ambulances. This finding may be due to higher administrative level’s involvement (E.g Health insurance agency and regional health bureau) in resource mobilization and management and use of common criteria in allocating resources.

Findings from this study also illustrate that there was no significant difference between the two groups in terms of service availability at facility level (non-LMG districts (65.0%) and LMG districts (65.7%)). This may be due to other interventions, such as, performance management through key indicators which encourage every district to ensure availability of services. Unlike this study, a study conducted in Kenya reported the significant effect of the LMG on service availability [22]. Another study conducted at facility level confirms that LMG training improved service availability by improving coordination, motivation, teamwork, commitment and relationship between parties [9].

This study proved the importance of performance management capacity building in improving service quality at the facility level. Data collected from LMG and non-LMG districts showed a significant relationship between management capacity and service quality. The service quality of non-LMG districts scored 54.2% and LMG districts scored 59.9%. This shows that capacity of managers at the district level highly determines the quality of service at the facility level. Program evaluations conducted in Zambia indicate that on-the-job mentoring to improve competencies of district management can result in improvements in quality of service by improving different aspects of leadership and management functions including planning and problem-solving skills [6]. A health care quality bulletin by the Federal Ministry of Health indicates the importance of problem identification and working towards improving quality of care [23].

Reading through the results from the study, it is good to note some of the limitations. The supervisions were made by project staff who may influence the observations. In addition, the analysis was only able to control the effect of background information which are available with the authors. A lack of baseline information about the study facilities was also a limitation of the study. In addition, the intervention was not “blinded” to the data collectors and this could have introduced bias into the results.

## Conclusion

District health offices are a critical structure to ensure the proper delivery of primary health care at the lower levels of the health system in Ethiopia. There are clear performance differences among various districts in the country despite there being no major differences among their characteristics. One of the reasons for this is the difference in the leadership skills among district health offices. As this study suggests, imparting the critical skills of leadership, management and governance functions coupled with problem solving skills through development of performance improvement projects and ongoing coaching contributes in improved district capacity, district structure and management practices, and quality of care.

## Supplementary Information

**Additional file 1.** Supportive supervision checklist categories

## Data Availability

The datasets used and/or analyzed during the current study are available from the corresponding author on reasonable request.

## Abbreviations

CBHI: Community-based health insurance
CI: Confidence Interval
DHIS: District Health Information System
EPI: Expanded Program of Immunization
FMOH: Federal Ministry of Health
HC: Health Center
HP: Health Post
IRB: Institutional Review Board
LMG: Leadership, Management and Governance
OALFA: Observe, Ask, Listen, Feedback, and Agreed
PHC: Primary Health Care
PRM: Performance Review Meeting
PSM: Propensity Score Matching
SD: Standard Deviation
SDG: Sustainable Development Goal
SMD: Standardized Mean Difference
SPSS: Statistical Package for Social Sciences
USAID: United States Agency for International Development
WHO: World Health Organization
