# Supplementary material for: Leadership, governance and management for improving district capacity and performance: the case of USAID transform: primary health care
Source: BMC Fam Pract. 2020 Dec 4;21:252. doi: 10.1186/s12875-020-01337-0 (PMC7718658; doi:10.1186/s12875-020-01337-0)
Supplement: Supplementary file 1 — Additional file 1. Supportive supervision checklist categories [file 12875_2020_1337_MOESM1_ESM.docx]

**Additional file 1** Supportive supervision checklist categories

| Categories |
| --- |
| **Management practices and structure** |
| District health office management committee has regular meetings and is transparent to staff |
| District health office self-assesses against management standards regularly |
| District health office has an updated training database |
| District health office develops HIS related improvement plans |
| District health office has management improvement projects that are successful |
| District health office conducted the last quarterly Integrated Supportive Supervision (ISS) |
| District health office conduced the last Quarterly Review Meeting (QRM) and /or EPAQ meeting |
| District health office uses strategies to identify pregnant women earlier on from within the community |
| District health office management has female members |
| Gender issues are discussed in district health office management meetings |
| District health office has a monitoring mechanism for compliance to clinical standards |
| District health office has implemented a mechanism for continuous management improvement |
| District health office conducts regular staff satisfaction surveys for the health facilities |
| District health office has staff recognition and motivation mechanisms |
| District health office creates forums for sharing and learning best practices for facilities |
| **Quality of services** |
| District health office has conducted medical audits of health facilities in the last quarter |
| District health office has a functional service quality assurance team |
| HFs use standard national guidelines while providing services |
| There was shortage of IMNCI, ICCM/CBNC drugs and supplies in the last one month |
| There was vaccine shortage in the past one month |
| There was HFs with stock-out of antimalarial drugs in the last three months |
| District health office regularly assess compliance to facility standards |
| **Resource mobilization and use** |
| District health office identifies gaps in human, financial and material resource in HFs |
| District health office facilitates auditing of PHC accounts |
| District health office has signed agreements with HFs to purchase health care services for CBHI members |
| District health office reimburses payments requested from HFs on a quarterly basis |
| There is practical AYHD responsive budgeting in the district |
| District health office has allocated a budget for district level SBCC activities |
| **Service availability** |
| Mean number of HCs |
| Mean number HCs providing LARC services |
| All HCs provide IMNCI services |
| All HPs provide ICCM/CBNC services |
| **District capacity** |
| District health office has a capacity to provide comprehensive FP (LARC) trainings |
| There is a functional PEMDSR team in the district |
| District health office provides IMNCI, ICCM/CBNC focused supervision |
| District health office has functional refrigerators |
| District health office have updated EPI monitoring charts |
| There are adequate numbers of trained YFS providers in the district |
| District health office has functional multisectoral forums that would respond to the AYHD issues |
| District health office has multisectoral coordination platform as per the national nutrition program |
| District health office has a trained person on gender analysis |
